# Improvement of Rice Agronomic Traits by Editing Type-B Response Regulators

**DOI:** 10.3390/ijms232214165

**Published:** 2022-11-16

**Authors:** Chuanhong Li, Chenbo Gong, Jiemin Wu, Linfeng Yang, Lei Zhou, Bian Wu, Liang Gao, Fei Ling, Aiqing You, Changyan Li, Yongjun Lin

**Affiliations:** 1 National Key Laboratory of Crop Genetic Improvement, Huazhong Agricultural University, Wuhan 430070, China; 2 Hubei Key Laboratory of Food Crop Germplasm and Genetic Improvement, Food Crops Institute, Hubei Academy of Agricultural Sciences, Wuhan 430064, China; 3 Hubei Hongshan Laboratory, Wuhan 430070, China

**Keywords:** rice breeding, CRISPR/Cas9, type-B response regulators, heading date, yield, quality

## Abstract

Type-B response regulator proteins in rice contain a conserved receiver domain, followed by a GARP DNA binding domain and a longer C-terminus. Some type-B response regulators such as RR21, RR22 and RR23 are involved in the development of rice leaf, root, flower and trichome. In this study, to evaluate the application potential of type-B response regulators in rice genetic improvement, thirteen type-B response regulator genes in rice were respectively knocked out by using CRISPR/Cas9 genome editing technology. Two guide RNAs (gRNAs) were simultaneously expressed on a knockout vector to mutate one gene. T_0_ transformed plants were used to screen the plants with deletion of large DNA fragments through PCR with specific primers. The mutants of CRISPR/Cas9 gene editing were detected by *Cas9* specific primer in the T_1_ generation, and homozygous mutants without *Cas9* were screened, whose target regions were confirmed by sequencing. Mutant materials of 12 *OsRRs* were obtained, except for *RR24*. Preliminary phenotypic observation revealed variations of various important traits in different mutant materials, including plant height, tiller number, tillering angle, heading date, panicle length and yield. The *osrr30* mutant in the T_2_ generation was then further examined. As a result, the heading date of the *osrr30* mutant was delayed by about 18 d, while the yield was increased by about 30%, and the chalkiness was significantly reduced compared with those of the wild-type under field high temperature stress. These results indicated that *osrr30* has great application value in rice breeding. Our findings suggest that it is feasible to perform genetic improvement of rice by editing the type-B response regulators.

## 1. Introduction

The two-component system was originally found in bacteria, which is involved in responses to hormones and biotic and abiotic environmental stimuli [1]. The simplest two-component system includes a histidine protein kinase and a response regulator (RR) [2,3]. The RR is the final protein in a two-component signaling transduction system, which receives the phosphate groups from histidine, and is widely present in bacteria, yeasts and plants [4]. The two-component system is widely involved in plant cellular processes, including hormone signaling, stress response, optical signaling and osmosensing [5,6,7]. Most RRs include the N-terminal conserved signal-receiver domain and the C-terminal variable regulator domain (also known as the output domain) [8,9]. Twenty-three RRs have been found in the model plant *Arabidopsis thaliana*, which can be classified into type-A (ARR3–ARR9, ARR15–ARR17), type-B (ARR1, ARR2, ARR10–ARR14, ARR18–ARR21), and type-C (ARR22 and ARR24) RRs based on their structures and sequences [10]. These RRs are involved in numerous processes of plant growth and development, such as rhythm clock, lateral root formation, meristem development, various hormones and stresses response, and so on [11].

The involvement of RRs in cytokinin signaling response and plant development regulation has been extensively studied in *Arabidopsis* [12,13]. Plant cytokinin signaling is actually a two-component system, which is composed of cytokinin receptors with histidine-kinase (HK) activity, histidine-containing phosphotransfer proteins (AHPs) and RRs [1]. HK receives cytokinin signaling by phosphorylation, passes it to HP, passes the phosphate group to different RRs genes in the nucleus, and then passes the cytokinin signal to different downstream genes through different RRs genes, and finally completes the CK signaling process [14]. *Arabidopsis* response regulators (ARRs) are divided into type-A, type-B and type-C ARRs according to their amino acid sequences and domains [10]. Type-A ARRs can be induced by cytokinin and provide negative feedback to regulate cytokinin signaling, including ARR3–9 and ARR15–17. Type-B ARRs encode transcription factors of DNA-binding domains (GARP domains, B motif), and the GARP domain can bind and regulate the transcriptional activation of type-A ARR genes; however, type-B ARRs cannot be induced by cytokinin. There are also numerous RR genes in rice (*Oryza sativa*) genome, including 13 type-A, 13 type-B and 2 type-C OsRRs. Type-A OsRRs contain a conserved receiver domain, a short N-terminus and C-terminus, while type-B OsRRs contain a conserved receiver domain followed by a GARP DNA binding domain and a longer C-terminus [15,16,17]. A previous study has demonstrated that OsRRs can bind to histidine phosphoric acid transfer proteins, and there are certain interactions between type-A and type-B OsRRs, confirming the presence of cytokinin signaling networks in rice similar to *Arabidopsis* [18]. It has been reported that type-A *OsRRs* can respond rapidly to cytokinin [19], and overexpression of the type-A *OsRR3* and *OsRR5* affects the response to cytokinin in rice [20]. In addition, the type-A *OsRR9* and *OsRR10* and type-B *OsRR22* can regulate the tolerance of rice to salt stress [21,22,23]. Three type-B genes, *OsRR21*, *OsRR22* and *OsRR23*, are also involved in the development of rice leaf, root, flower and trichome [24].

The defense system, composed of clustered regularly interspaced short palindromic repeats (CRISPR) and their associated proteins (Cas), is widely present in bacteria and archaea [25,26,27,28]. This adaptive immune system is a CRISPR region of the microbes that captures small pieces of foreign DNA and integrates them into the CRISPR region of its own genome, then binds to the invading foreign DNA through crRNA base-paring, and finally cleaves it with the Cas nuclease [25,26,29,30]. In 2012, two almost simultaneous reports proved that precise cleavage of DNA can be achieved by small fragments of RNA and the Cas9 protein in vitro, signifying the start of an era of CRISPR/Cas gene editing [31,32]. In 2013, there were three research papers reporting the use of Cas9 and gRNA for precise gene editing in human cell lines [33,34,35]. The CRISPR/Cas9 system binds to the target sequence by gRNA, and then Cas9 produces a flat-ended double-strand break (DSB) by a cleavage upstream of the PAM sequence, resulting in base deletion, insertion and replacement mutations during repairing these double-stranded broken DNA through homologous recombination and non-homologous recombination [33,35]. After 2013, the development, engineering and application of CRISPR/Cas gene editing tools have entered a period of explosive growth, and type II Cas9 and type V Cas12 variants with different nucleic acid endonuclease activities have been successively developed and applied to the CRISPR/Cas gene editing system, greatly expanding the range of gene editing target sequences (more diverse PAM sequences) and improving the specificity of DNA cleavage [36]. In addition, modification of gRNAs can also reduce the off-target rate [37].

Genetic variation is the basis for genetic improvement, and breeding is a process of creating and selecting genetic variations [38]. Throughout the long history of plant breeding, there are generally four main means of breeding: cross breeding, mutation breeding, transgenic breeding and genome editing breeding [39]. Traditional cross breeding and mutation breeding are time-consuming and labor-intensive; Transgenic breeding can greatly accelerate the breeding and confer new traits to crops, but genetically modified (GM) crops are subjected to strict government regulations, so their development is of high costs; There are currently few types of commercially grown GM crops, and the negative public opinion on their safety has severely limited their huge potential [38]. Genome editing can quickly and accurately modify genomic information to achieve breeding goals, exhibiting great potential in breeding [38,40]. The use of CRISPR/Cas gene editing technology to delete or mutate genes with negative effects on the trait of interest or introduce genetic factors favorable to breeding traits to achieve genetic improvement has greatly improved the traits such as yield, quality, pest resistance and herbicide resistance [41].

Here, to study the application potential of type-B RR genes in rice genetic improvement, CRISPR/Cas9 genome editing technology was used to knock out 13 type-B *OsRRs*. Two gRNAs were simultaneously expressed on a knockout vector to mutate one gene. T_0_ transformed plants were screened for the deletion of large DNA fragments through PCR with specific primers. The mutant materials of CRISPR/Cas9 gene editing were detected by *Cas9* specific primer in T_1_ generation, and the homozygous mutants without *Cas9* were screened; besides, their target regions were sequenced and confirmed. A total of 12 *OsRR* mutant materials were obtained. Preliminary phenotypic observation revealed that many traits varied among different mutant materials, including plant height, tiller number, tillering angle, heading date, panicle length and yield. We then specifically examined the *osrr30* mutant in the T_2_ generation. As a result, the *osrr30* mutant plants showed an 18 d delay of heading date but an about 30% increase in yield, and a significant reduction of chalkiness compared with the wild-type (WT) under field high temperature stress, indicating that *osrr30* is of great breeding application value. In addition, our results demonstrated the feasibility of rice genetic improvement by editing the type-B *OsRRs* in rice.

## 2. Results

### 2.1. Domain and Phylogenetic Tree Analysis of Rice Cytokinin Response Regulators

**Figure 1:**
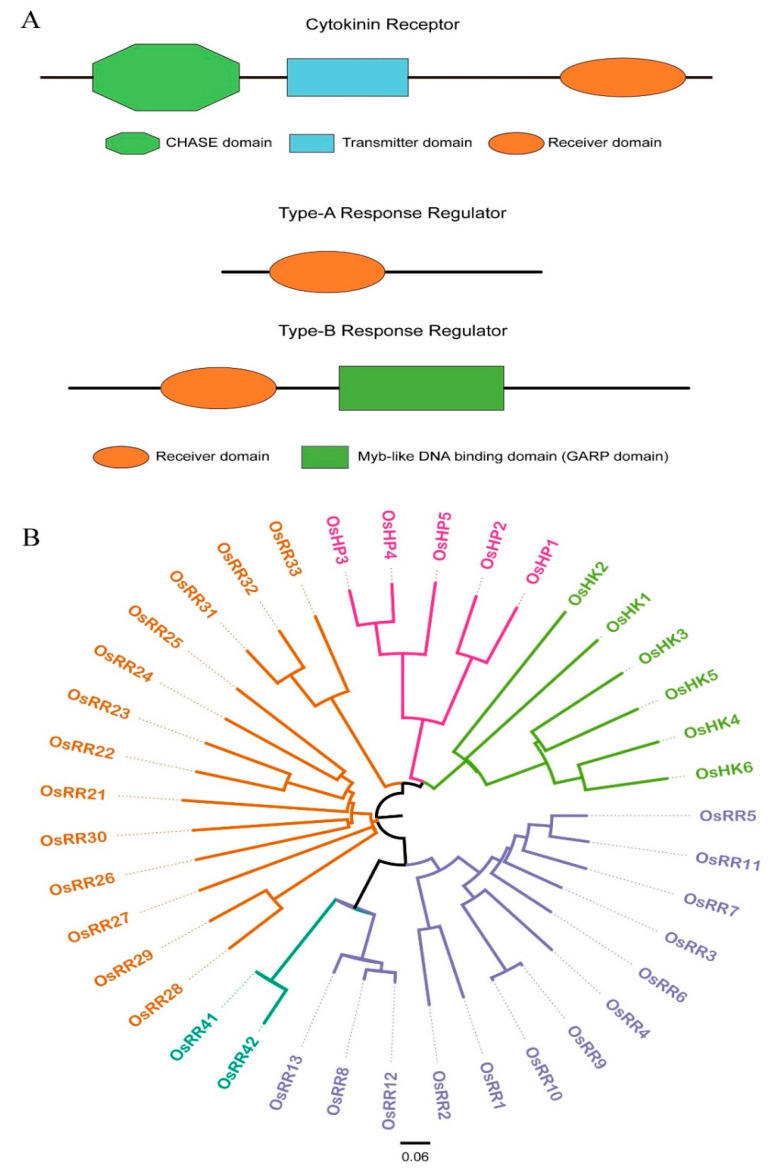
Domain and phylogenetic tree of response regulators (RRs). (**A**). Structures of type-A and type-B RRs. (**B**). Phylogenetic analysis of RRs in rice. The analysis was conducted using complete amino acid sequences with the neighbor-joining method implemented in MEGA X. Purple, orange and blue represent type-A, type-B and type-C RRs, respectively.

RRs play an important role in the process of cytokinin signal transduction. To study the functions of *OsRRs* for genetic improvement, we searched for the relevant information of *OsRR* genes in rice genome from the UniprotKB and TIGR databases, including *OsRR* name, TIGR accession number, genotype, distribution in chromosomes, length of coding sequence (CDS) and number of exons, which provide a basis for homology alignment of OsRR protein sequences. There were 28 *OsRRs* in rice genome, including 13 type-A *OsRRs* (*OsRR1*-*13*), 13 type-B *OsRRs* (*OsRR21*-*33*) and 2 type-C *OsRRs* (*OsRR41*-*42*) (Figure 1B). In general, the CDS of type-B *OsRRs* was longer than that of type-A *OsRRs*, because type-B OsRRs not only contain the conserved D-D-K motif of type-A *OsRRs* (receiver domain), but also harbor an additional Myb motif (motif) that can bind to type-A *OsRR* promoters (Figure 1A). In addition, there were two type-C *OsRRs* (*OsRR41* and *OsRR42*). In addition to these 28 relatively well-defined *OsRR* genes, there were eight predicted *OsRRs* genes (Supplementary Material S1).

The type-B OsRRs studied here had an N-terminal receiver domain, and their central part had the GARP DNA binding domain and a longer-variable C-terminus. According to a previous report, there are 13 type-B *OsRRs* in rice genome, and the gene information is presented in Table 1. The information of amino acid sequences of type-B OsRRs was obtained from the Rice Genome Annotation Project (http://rice.uga.edu/, accessed on 7 October 2022) [42] and Rice Annotation Project Database (https://rapdb.dna.affrc.go.jp/, accessed on 7 October 2022) databases [43]. OsRRs phylogenetic relationship tree was constructed by using the Neighbor-Joining method of MEGA X [44]. Sequence alignment of OsRRs demonstrated that they can be clearly divided into two major branches, namely type-A (orange) and type-B (blue).

### 2.2. Sequence Alignment and mRNA Expression Patterns of Type-B OsRRs in Rice

**Figure 2:**
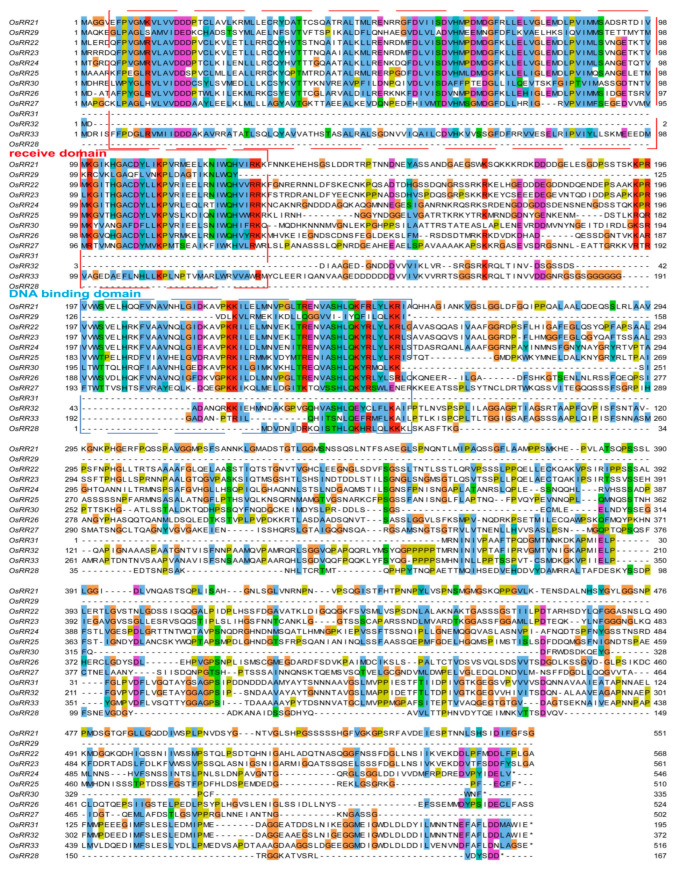
Multiple sequence alignment of type-B OsRRs. Red box indicates receive domain; blue box indicates DNA binding domain.

Alignment of the 13 type-B OsRRs in rice demonstrated that except for OsRR28, OsRR31 and OsRR32, the remaining 10 OsRRs had a highly conserved N-terminus, probably because the N-terminus is mainly the receiver domain with relatively conserved function, and the conservation degree of the C-terminus is relatively low (Figure 2). It is possible that the C-terminus is mainly a DNA-binding domain, which requires different type-B OsRRs to target different downstream targets. The lower conservation degree of C-terminus endows type-B OsRRs with physiologically different functions.

**Figure 3:**
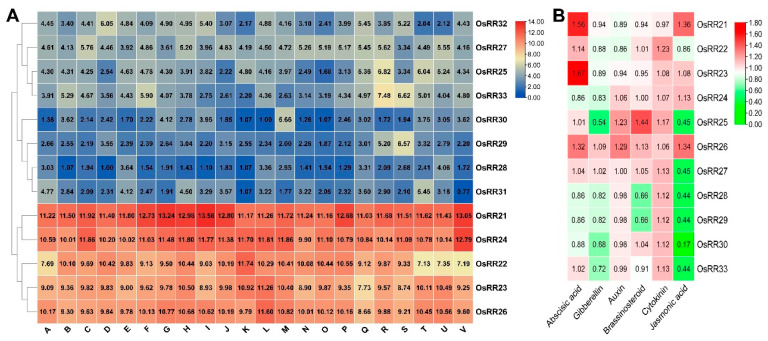
Expression pattern analysis of type-B *OsRRs* in rice. (**A**). Expression pattern analysis of type-B *OsRRs* throughout the growth period (A: germinating seed harvested at 72 h post imbibition. B: Light and dark grown plumules harvested at 48 h after germination. C: Light and dark grown radicles harvested at 48 h after germination (radicle 1, radicle 2). D: Three-day-old seedling. E: Shoot of seedlings with three two tillers. F: Roots of seedlings with three tillers. G: Leaf tissues from plants with panicles shorter than 1 mm. H: Sheath tissues from plants with panicles shorter than 1 mm. I: Leaf tissues from plants with panicles between 40 and 50 mm. J: Sheath tissues from plants with panicles between 40 and 50 mm. K: Panicle (40 to 50 mm). L: Stem tissue at 5 d before flowering. M: Leaf tissues at 5 d before heading. N: Stem tissue at the heading stage, O: Panicle at the heading stage. P: Palea/lemma at 1 d before flowering. Q: Stamen at 1 d before flowering. R: Spikelet at 3 d post anthesis. S: Endosperm at 7 d post anthesis. T: Endosperm at 14 d post anthesis. U: Endosperm 21 d post anthesis. V: Flag leaf at 14 d post anthesis). (**B**). Expression pattern analysis of rice type-B *OsRRs* in response to different hormone treatment.

All the 13 type-B *OsRRs* were constitutively expressed throughout the growth period as indicated by the expression profiles. Among them, *OsRR21*, *OsRR*22, *OsRR23*, *OsRR*24 and *OsRR26* were highly expressed in different tissues; four genes, including *OsRR25*, *OsRR27*, *OsRR32* and *OsRR33*, had moderate expression levels in different tissues; while *OsRR28*, *OsRR29*, *OsRR30* and *OsRR31* had lower expression levels in different tissues. Two-component signaling systems have been found to be involved in plant hormone signaling [45], but relatively little research has been reported that type-B *OsRRs* response to various hormone treatment in rice. In order to further verify the expression of type-B *OsRRs* after hormone treatment, we analyzed the expression levels of 10 type-B *OsRRs* in the shoots of rice seedlings at 12 h after treatment with six hormones by using the information in the public database RiceXPro (http://ricexpro.dna.affrc.go.jp/, accessed on 7 October 2022) [46]. As a result, type-B *OsRRs* showed weak responses to various hormone treatments, and only a few family members were down-regulated after JA treatment, particularly *OsRR30*, whose expression was decreased by more than 80% at 12 h of JA treatment (Figure 3).

### 2.3. Gene Editing of Type-B OsRRs by CRISPR/Cas9 System

**Figure 4:**
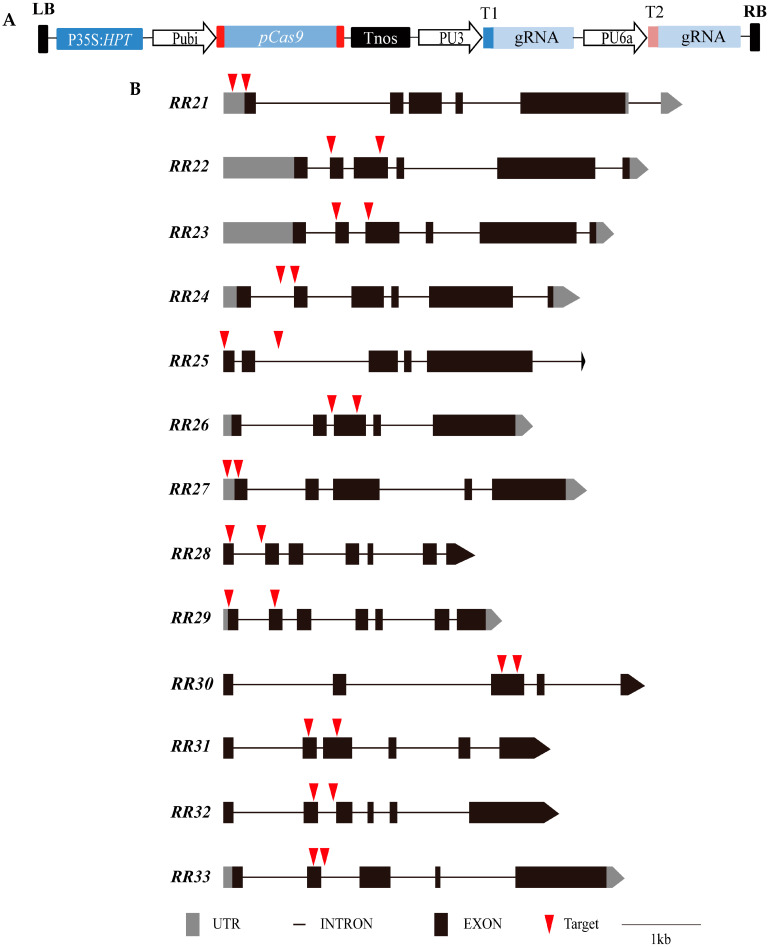
Diagram of CRISPR/Cas9 construction and distribution of targets on *OsRRs*. (**A**)**.** Diagram of CRISPR/Cas9 construction. (**B**). Distribution of targets on type-B *OsRRs*.

To explore the roles of type-B *OsRRs* in rice growth and development and evaluate their application potential in rice genetic improvement, CRISPR/Cas9 genome editing technology was used to respectively knock out 13 type-B *OsRRs* in rice. Then, two gRNAs were simultaneously expressed on one knockout vector to mutate one gene (Figure 4), which could help the preliminary screening of T_0_ generation transgenic seedlings by PCR directly and reduce the screening cost.

**Figure 5:**
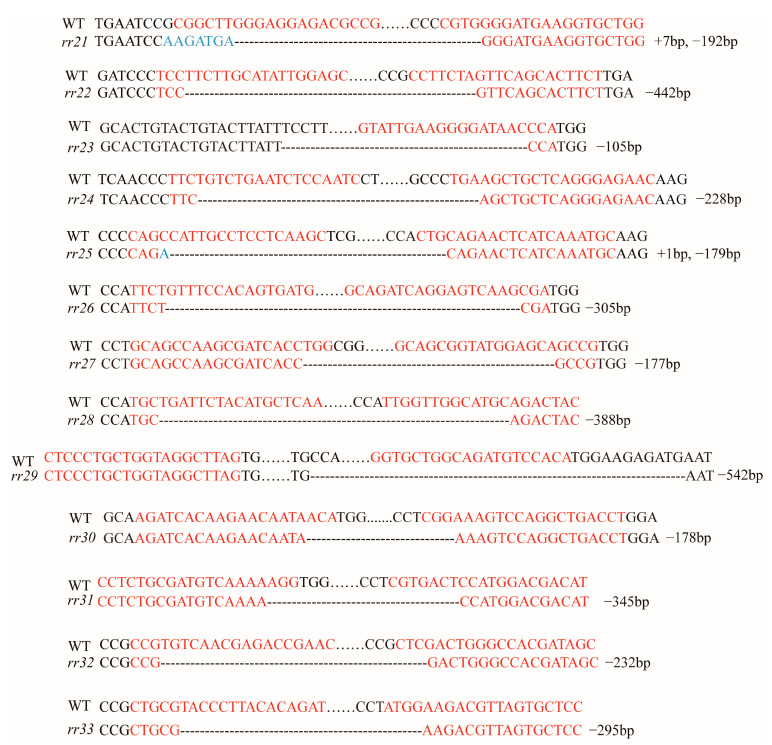
Mutation of type-B *OsRRs* in detail. The target sequences were red characters, and --- indicates the bases deleted in the mutants.

Transformed plants of the T_0_ generation were detected by PCR with specific primers, and plants with the deletion of large DNA fragments were screened out. The specific information of the PCR detection results is shown in Table 2. Among them, nine *OsRRs* had homozygous deletion of large fragments in the T_0_ generation, and the remaining four *OsRRs* had heterozygous lines with deletion of large fragments, which were detected in the T_1_ generation to obtain homozygous lines with large fragment deletions. All homozygous lines were then sequenced and verified (Figure 5); however, *osrr24* failed to set seeds as previously reported [47], and, thus, the *osrr24* homozygous mutant was not further investigated in this study. In addition, we also tested the *Cas9* in the T_1_ generation of CRISPR/Cas9 gene-edited plants, and 12 *OsRR* mutants were found to homozygous of large fragment deletion and did not contain *Cas9*. T_2_ generation plants were planted in the field for further observation of agronomic traits.

### 2.4. Field Agronomic Traits of T_2_ Generation Mutants of Type-B OsRRs

**Figure 6:**
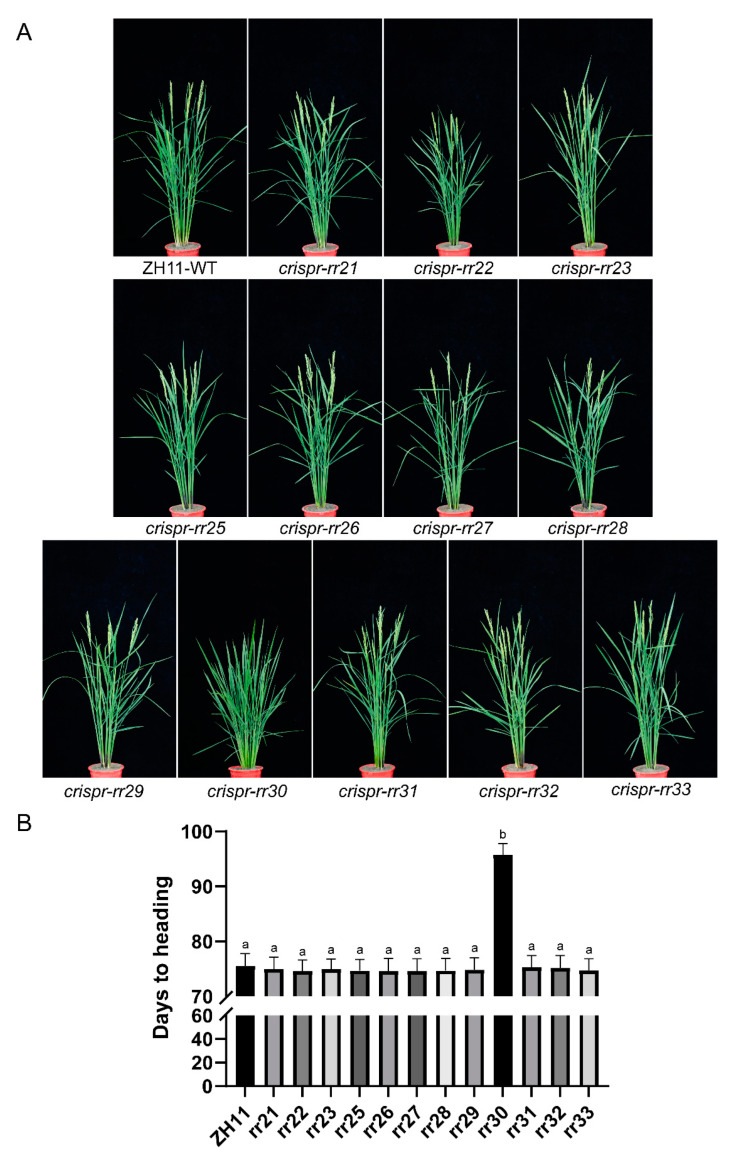
Heading date of type-B *OsRR* mutants. (**A**). Phenotypes of WT and mutants at the heading stage. (**B**). Days of heading in WT and mutants (n = 20). Values are presented as mean ± standard deviation (SD). The “a and b” respectively represent the differences determined by least significant difference (LSD) method with *p* = 0.05, the same letter indicates that there is no significant difference from each other.

Transformed materials of the mutant T_2_ generation were planted in a Wuhan experimental field (sown in mid-May under natural long-day conditions) to investigate their agronomic traits in the field. Twenty plants of each mutant material were examined and observed in the field for agronomic traits. Compared with the WT, the *osrr22* mutant showed lower plant height and shorter leaves and ears; *osrr25* was slightly lower in plant height; and *osrr31* and *osrr32* had a more compact plant architecture with a smaller tiller angle (Figure 6A). We also investigated the heading date of all mutant materials. Surprisingly, all mutants showed no significant difference from the WT except for the *osrr30* mutant, which exhibited a significant delay in the heading date by about 18 d (Figure 6B) but a good growth performance (Figure 6A). Furthermore, in the artificial climate chamber, a significant delay in heading was also observed for *osrr30* under both short-day (10 h light) and long-day (14 h light) conditions.

### 2.5. OsRR30 Mutation Delays Heading while Improves Rice Yield and Quality

**Figure 7:**
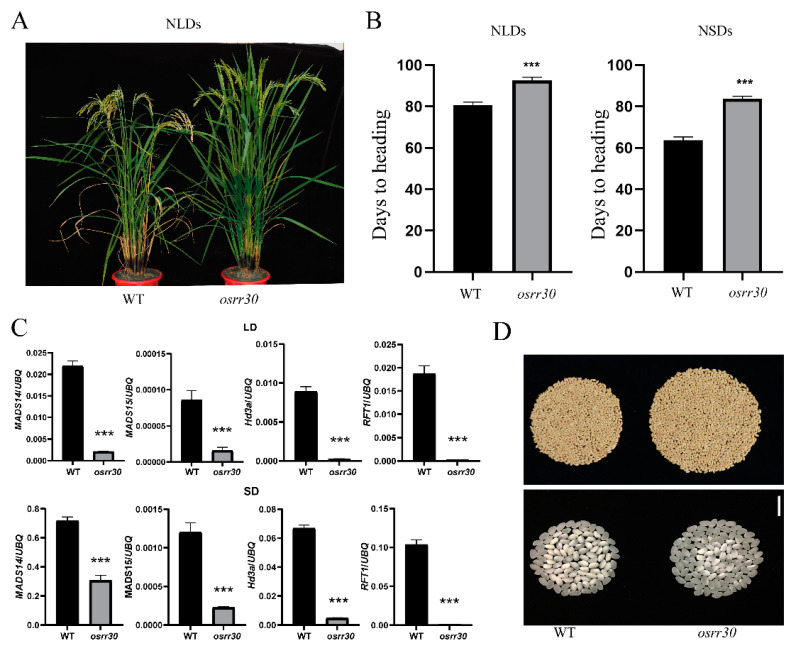
Delay of heading and improvement of yield and quality of *osrr30* mutant. (**A**). Gross morphology of *osrr30* and WT. (**B**). Heading date of *osrr30* and WT under natural long-day conditions (NLDs) and natural short-day conditions (NSDs) in Wuhan. (**C**). Transcription levels of flowering-related genes in WT and *osrr30* plants under LD (upper) and SD (down) conditions. (**D**). *osrr30* showed improvement of yield and reduction of chalkiness rate. Student’s *t* test, *** indicates *p* < 0.001.

Under field conditions, the *osrr30* mutant exhibited a significant delay of heading under both short-day (delay by 18 d) and long-day (delay by 20 d) conditions, as well as taller plant height compared with the WT (Figure 7A,B). A further examination of the expression of flowering-related genes (*MADS14*, *MADS15*, *Hd3a* and *RFT1*) revealed that these genes were significantly down-regulated in *osrr30* under both long-day and short-day conditions (Figure 7C).

The agronomic traits of *osrr30* mutant and WT were investigated under natural long-day conditions in Wuhan. The number of tillers and 1000-grain weight of the mutants were not significantly different from those of the WT, but the mutants had significantly higher plant height, panicle length, seed setting rate, grains per panicle and yield per plant. Compared with that of the WT, the final yield of the mutant was increased by 27.9% and reached 33.58 g per plant, which could be mainly ascribed to the increase in panicle length and number of grains per panicle (Table 3). In addition, the *osrr30* mutant showed significant reduction of grain chalkiness and improvement of grain quality relative to WT (ZH11), which generally showed high chalkiness when planted in the same experimental field (Figure 7D). These results indicated the great application potential of this gene in practical production.

## 3. Discussion

### 3.1. Characteristics of Type-B OsRRs

Unlike type-A OsRRs with only one N-terminal signal-receiver domain, type-B OsRRs have a DNA binding domain GARP (a typical MYB transcription factor) in addition to an N-terminal receiver domain. Most type-B OsRRs are localized in the nucleus [15]. Additionally, their transcriptional activation regions contain large amounts of proline and glutamate, implying that they might be transcriptionally activated throughout the signal transduction pathway [48]. Some studies have suggested that type-B OsRRs can bind to the promoter of type-A OsRRs and thus activate their expression, indicating that type-B OsRRs may function upstream of type-A OsRRs [49]. Type-B *OsRRs* are expressed in all tissues of rice, probably because cytokinin is widely distributed in the organs of higher plants, and all organs of rice can sense the cytokinin signal. Although all type-B *OsRRs* were constitutively expressed, distinct differences can be observed in their expression levels, among which *OsRR21*, *OsRR22*, *OsRR23*, *OsRR24* and *OsRR26* were highly expressed in different tissues throughout the growth period. *OsRR25*, *OsRR27*, *OsRR32* and *OsRR33* showed moderate levels of expression, while *OsRR28*, *OsRR29*, *OsRR30* and *OsRR31* exhibited low expression levels in different tissues during the whole growth period. Unlike type-A *OsRRs*, whose expression was reported to be induced by hormones, all type-B *OsRRs* showed weak responses to hormone treatments, and only a few members exhibited down-regulated expression in the aerial part after JA treatment, such as *OsRR30*, whose expression was down-regulated by more than 80% after 12 h of JA treatment.

We suspect that these expression patterns throughout the growth period and after hormone treatment may not be the actual and precise expression patterns of type-B *OsRRs*, since there has been increasing evidence showing that the initiation of plant growth and development often starts from a small number of primary meristematic cells. More precise expression assays (in situ hybridization and single-cell sequencing) are required to obtain the exact expression pattern of type-B *OsRRs*. Phenotypic observation of 12 homozygous T_2_ plants of type-B *OsRRs* revealed that the *osrr22* mutant had dwarf plant heights as well as shorter leaves and panicles. The *osrr25* mutant also showed a slight reduction of plant height relative to the WT, and the *osrr31* and *osrr32* mutants were more compact in plant architecture with smaller tiller angles. The *osrr30* mutant had an obvious delay of heading but good growth performance, which was not found in other *osrr* mutants. There may be redundancy in the function of some type-B *OsRRs* [24,50], and their functions need to be further verified by multi-mutation.

### 3.2. Application of Gene Editing in Rice Breeding

It is estimated that the global population will reach 9 billion by 2050, and the demand for food will increase significantly [51,52]. Breeders need to pay more attention to yield traits, and there will be higher demands for breeding techniques [53]. Currently, cross breeding, mutation breeding and transgenic breeding are the main methods of genetic improvement in modern agriculture. Traditional cross breeding requires many years of cross selection for the introduction of desirable alleles and improvement of genetic diversity. Mutation breeding uses chemical mutagenesis and physical radiation to introduce genetic variations, in which the mutation is random and also a long time is required for selection. Transgenic breeding can rapidly transfer foreign genes into elite varieties to obtain desired traits, but its commercial application is limited by lengthy and expensive regulatory evaluations and public concerns [38,54]. Gene editing technologies have been developed to efficiently and rapidly introduce precise and predictable variations into the genome to obtain desired traits [38,40]. At present, the CRISPR/Cas editing system is applied to genetic improvement and plant breeding research through means such as gene knockout, gene knock-in and substitution, base editing, regulation of gene expression, and high-throughput mutant library [40,55]. Gene editing has been applied to the de novo domestication of wild crops [56,57,58], and the rapid domestication of orphan crops [59]. So far, most gene knockouts using cas9 are random mutations of single target sites, which will lead to random insertion, deletion or mutation of the target site during the repair process. However, the majority of single site gRNA-mediated mutations are deletions, insertions or mutations of a few bases or even a single base. These mutations in most cases cannot be detected by PCR directly and can only be identified by large-scale sequencing, which is time-consuming, labor-intensive and costly. The double gRNA site-directed deletion for the target site used in this study can easily determine whether the mutation is homozygous or heterozygous by PCR. In this way, the desired transgenic plants with homozygous mutation can be accurately and rapidly selected for sequence verification, which reduces the workload and cost of sequencing, improves the accuracy of mutant plant selection, and also facilitates the genotype identification of progeny.

### 3.3. Application Potential of OsRR30 in Rice Breeding and Strategies for Further Improvement

Under field conditions, the *osrr30* mutant showed a significant delay of heading under both short-day and long-day conditions, as well as accumulation of more biomass. Subsequent detection of the expression of the flowering-related genes *Hd3a*, *RFT1, MADS14* and *MADS15* demonstrated that these genes were significantly down-regulated in *osrr30* under both long-day and short-day conditions. In addition, the *osrr30* mutant exhibited significant improvement of yield (by 27.9% relative to the WT) and quality with a prolonged growth period, indicating that the mutation of this gene has great potential for production application. *OsRR30*/*Ehd1* has been mainly studied and reported as a key regulator of flowering specific to rice in the early stage, and relatively little research has occurred in terms of yield. Our study confirmed that the *OsRR30*/*Ehd1* mutation increased yield associated with a delay in heading [60]. However, the delay of heading for the mutant was as long as 18 d and 20 d under long-day and short-day conditions, respectively. In actual production, a too significant delay of heading may largely hinder the application of this gene. In the future, some other editing strategies may be used to fine-tune the function of *OsRR30* instead of deleting its function directly. For example, the promoter may be edited to achieve an appropriate reduction of expression to confer the mutant with a more reasonable delay of heading. It has been reported that the tiling deletion of the promoter of *IPA1* could screen for the mutants that could balance tiller number and size and thereby increase the yield [61]. In addition, CRISPR-Cas can be employed to edit the coding region of *OsRR30* to obtain weaker mutations for the appropriate heading date.

## 4. Materials and Methods

### 4.1. Plant Materials and Growth Conditions

The japonica rice variety ZH11 (*Oryza sativa* L. ssp. Geng/Japonica cv. Zhonghua 11) was used as the wild type and recipient for genetic transformation. All materials were planted in the experimental farm of Huazhong Agricultural University (30.4° N, 114.2° E) in Wuhan, and Lingshui, Hainan Province (18.5° N, 110° E). The rice material was also planted in an artificial climate box (B8, Eshengtaihe Ctrl Tech, Kunming, China) with long-day conditions of 14 h light at 30 °C/10 h dark at 25 °C, and short-day conditions of 10 h light at 30 °C/14 h dark at 25 °C.

### 4.2. Vector Construction and Plant Transformation

The Cas9 plant expression vector (pYLCRISPR/Cas9Pubi-H) and the sgRNA expression vector (pYLgRNA) were provided by Prof. Yao-Guang Liu of the South China Agricultural University. To construct the rice type-B response regulator CRISPR/Cas9 vector, two targets were designed at the 5′-end or intermediate region of each gene, and the two targets were constructed onto one CRISPR/Cas9 vector (pYLCRISPR/Cas9-MH), and the two sgRNA expression cassettes were driven by the rice U3 and U6 snRNA promoters, respectively. The targets were selected using CRISPR-P2.0 software (http://crispr.hzau.edu.cn/CRISPR2/, accessed on 7 October 2022) [62], and the target primers are shown in Supplementary S2. CRISPR/Cas9 vector construction was performed in reference to previous literature [63]. The vector was introduced into *Agrobacterium tumefaciens* strain EHA105 by electroporation and subsequently introduced into ZH11 callus by *Agrobacterium*-mediated transformation as previously described [64].

### 4.3. Phenotype Investigation and Evaluation of Agronomic Traits

The seeds of mutants and wild type ZH11 were sown at the same time in the field. Twenty-six days after sowing, the seedlings were transplanted into the same field site. Mutants and ZH11 were planted in the field with a line distance of 16.5 cm and a row distance of 19.8 cm. Heading dates under different conditions were recorded as the number of days from germination to the emergence of the first panicle (20 plants per line). Plant height was measured from the ground to the top of the tallest tiller of the plant before harvesting. Ten plants were randomly selected from each plot and their agronomic traits were investigated. Grains were harvested individually within each line and dried under sunlight for 5 d. Then, the yield and yield component traits, including panicles per plant, panicle length, filled grains per panicle, grain filling rate, 1000-grain weight, and yield per plant, were measured.

### 4.4. Molecular Characterization of the Mutant Plants

To identify the type-B *OsRR* mutants, the genomic DNA of all transgenic herbicide-resistant T_0_ plants was first examined by PCR using primer pairs flanking the designed target sites. Only plants with large DNA fragment deletions were selected for further analysis. Genomic DNA of T_1_ transgenic plants was used as the template to perform PCR amplification using *Cas9* specific primer (Cas9F: CACGAGGTCCGACAAGAACA and Cas9R: ACCTTGCGAACAGTAGCGAA), and the *Cas9* free plants were selected for further study. To examine the deletion details, PCR fragments amplified with the specific primer pairs surrounding the designed target site from *Cas9*-free homozygous plants were sequenced by the Sanger sequencing [65].

### 4.5. RNA Isolation and RNA Expression Analysis

Total RNA was extracted from leaves at 49 d after sowing using the TRIzol reagent (Invitrogen, Waltham, MA, USA). First-strand cDNA was synthesized using the SuperScript III Reverse Transcriptase (Invitrogen, USA). qRT-PCR was conducted with the FastStart Universal SYBR Green Master (ROX) (Roche, Basel, Switzerland) on an Applied Biosystems QuantStudioTM6 Flex System (Applied Biosystems, Waltham, MA, USA). The rice *ubiquitin* gene was used as an internal control for normalization.

### 4.6. Sequence and Expression Analysis of Type-B Response Regulators

The sequences of rice type-B OsRRs were obtained from the Rice Genome Annotation Project (http://rice.uga.edu/, accessed on 7 October 2022), and the Rice Annotation Project Database (https://rapdb.dna. affrc.go.jp/, accessed on 7 October 2022). Phylogenetic analyses were conducted in MEGA X using the neighbor-joining method with support values based on 1000 bootstrap replications. Gene expression data throughout the life cycle of rice were obtained from the NCPGR database (http://www.ncpgr.cn/web/, accessed on 7 October 2022) [66]. Gene expression data after hormone treatment were obtained from the RicXPro database (http://ricexpro.dna.affrc.go.jp/, accessed on 7 October 2022). Heat map of gene expression was constructed by using TBtools [67].

## 5. Conclusions

In this study, we analyzed the expression patterns of type-B *OsRRs* in the entire life cycle of rice and their responses to hormone treatments. CRISPR/Cas9 was used to create mutants of all type-B *OsRRs*, which exhibited variations in agronomic traits. The main findings are as follows. (1) All 13 type-B *OsRRs* are constitutively expressed in the whole growth period, but there are wide variations in their expression levels. (2) All type-B *OsRRs* have weak responses to hormone treatments, and only a few family members were down-regulated after JA treatment. (3) The *osrr30* mutant, which showed a significant delay of heading date, has a significant reduction of chalkiness rate, improvement of quality, and increase in yield, indicating that the gene has great application potential in actual production. (4) The created mutants with changes in plant architecture, panicle shape and leaf shape provide an important resource for genetic improvement of rice in the future.

## Figures and Tables

**Table 1:** Information of type-B *OsRRs* in rice.

Gene Name	MSU ID	RAP ID	Chromosome	Length (CDS)	Exon
*OsRR21*	LOC_Os03g12350	Os03g0224200	3	2073	5
*OsRR22*	LOC_Os06g08440	Os06g0183100	6	2088	6
*OsRR23*	LOC_Os02g55320	Os02g0796500	2	2064	6
*OsRR24*	LOC_Os02g08500	Os02g0182100	2	1878	6
*OsRR25*	LOC_Os06g43910	Os06g0647200	6	2070	6
*OsRR26*	LOC_Os01g67770	Os01g0904700	1	1746	5
*OsRR27*	LOC_Os05g32880	Os05g0395600	5	1860	5
*OsRR28*	LOC_Os04g28160	Os04g0349100	4	1140	7
*OsRR29*	LOC_Os04g28130	Os04g0348800	4	1170	7
*OsRR30*	LOC_Os10g32600	Os10g0463400	10	1023	5
*OsRR31*	LOC_Os08g35650	Os08g0458400	8	1455	6
*OsRR32*	LOC_Os08g17760	Os08g0279900	8	1710	6
*OsRR33*	LOC_Os08g35670	Os08g0458600	8	1842	5

**Table 2:** Summary of genome editing results in T_0_ transgenic rice by PCR.

Gene	No. of T_0_ Plants	Targeted Deletion	
		Homozygous (No.%)	Heterozygous (No.%)
*OsRR21*	75	24 (32%)	27 (36%)
*OsRR22*	35	1 (3%)	12 (34%)
*OsRR23*	48	0 (0%)	7 (15%)
*OsRR24*	54	0 (0%)	14 (26%)
*OsRR25*	49	0 (0%)	6 (12%)
*OsRR26*	51	1 (2%)	16 (31%)
*OsRR27*	43	1 (2%)	13 (30%)
*OsRR28*	64	6 (9%)	16 (25%)
*OsRR29*	40	0 (0%)	6 (15%)
*OsRR30*	78	6 (8%)	25 (32%)
*OsRR31*	45	3 (7%)	6 (13%)
*OsRR32*	39	2 (5%)	5 (13%)
*OsRR33*	33	1 (3%)	5 (15%)

**Table 3:** Agronomic performance of WT and *osrr30* plants.

Lines	Heading Date (d)	Plant Height (cm)	Tillers per Plant	Panicle Length (cm)	Filled Grain Rate (%)	1000-Grain Weight (g)	Grains per Panicle	Yield per Plant (g)
WT	80.4 ± 2.0	101.8 ± 2.0	9.7 ± 0.8	22.74 ± 0.33	79.74 ± 1.61	23.45 ± 0.43	145.3 ± 5.9	26.26 ± 1.05
*osrr30*	92.2 ± 2.2 ***	105.8 ± 1.9 ***	10.0 ± 0.8 ns	25.51 ± 0.50 ***	82.26 ± 1.48 **	24.04 ± 0.98 ns	170.5 ± 7.3 ***	33.58 ± 0.9 ***

The plants were grown under natural field conditions in the experimental station of Huazhong Agricultural University, Wuhan, China (30.4° N, 114.2° E), mid-May 2019. Values are mean ± standard deviation (SD) for data collected from 10 plants for each plant type. Student’s *t* test, ns indicates not significant, ** indicates *p* < 0.01, *** indicates *p* < 0.001.

## Data Availability

Not applicable.

## Supplementary Materials

The following supporting information can be downloaded at: https://www.mdpi.com/article/10.3390/ijms232214165/s1.
