# Supplementary material for: Improvement of Rice Agronomic Traits by Editing Type-B Response Regulators
Source: Int J Mol Sci. 2022 Nov 16;23(22):14165. doi: 10.3390/ijms232214165 (PMC9698459; doi:10.3390/ijms232214165)
Supplement: Supplementary file 1 [file ijms-23-14165-s001.zip › ijms-1986700-Supplementary/Supplementary 1.pdf]

>OsRR1 A-type response regulator, Cytokinin signaling
MEGGRGVTRVLLVDDSPVDRRVVQLLLSSSACAGSFHVIAVDSAKKAMEFLGLKEEGKE
QAIDMVLTDYCMPEMTGYELLKAIKALSPLKPIPVIVMSSENEPQRISRCMNAGAEDFIVK
PLQSKDVQRLRNCSPANTQCCDAGSDGKPPLLLLPSDHVVVDATAASPPPPPSRRRAHFAG
VAMVLHSSSVELSHYFPFLFKFILLVYAILCLGELLHRWSNGCFLNLWCA*
>OsRR2 A-type response regulator, Cytokinin signaling
MGAEAVRVLVVDDSPVDRRVVELLLRAHCGGGGGAAAGEAAPFHVTAVDSGKKAMELL
GRRRGDRDHLTPSSPAAAAAANDQAIDIVLTDYCMPEMTGYDLLKAIKALGSPNPIPVVV
MSSENEPQRISRCLTAGAEDFILKPLKMNDVQRLRKCSGATRPKSAVAGDDDRCCNTAKK
AAAAAAATPEQQQQQQRSSHLAGLAMVMNASSFEVSHYFQLIFKLILLAYAVLCLSQLLH
RWSNGSSLLSLWCA*
>OsRR3 A-type response regulator, Cytokinin signaling
MSTKTVPEPEPHVLAVDDSIVDRTVISRLLRSSKYRVTTVDSGKRALEVLSLDRNVHMIIT
DYCMPEMTGFDLLKRVKESAELKEIPVVLMSSENSPTRIRRCLEEGAEDFLIKPVRPSDVS
RLCNRVIMK*
>OsRR4 A-type response regulator, Cytokinin signaling
MTVVDAESRFHVLAVDDSLIDRKLIEMLLKNSSYQVTTVDSGSKALELLGLRDEGDDSSS
SPSSSSPDHQEIDVNLIITDYCMPGMTGYDLLKRVKGSSSLKDIPVVIMSSENVPARINRCL
EDGAEEFFLKPVKLADMKKLKSHLLKRKQQLPMAAAAPDKPPHKPDEAAASAAAIAEA
ATAQTDGIISDCSCSGSSKRKAAAMEQEVISSPDQRTKPRLSSTSSGLAVET*
>OsRR5 A-type response regulator, Cytokinin signaling
MATCRSRGVERGGAPHVLAVDDSSVDRAVISGILRSSQFRVTAVDSGKRALELLGSEPNVS
MIITDYWMPEMTGYELLKKVKESSRLKEIPVVIMSSENVSTRINRCLEEGAEDFLLKPVQP
SDVSRLCSRVLR*
>OsRR6 A-type response regulator, Cytokinin signaling
MAAAAQAPAAAKVVVATSPRAGGGGGGGGDRKVVPVVVAAAAGDEAQSEMHVLAVDD
SSVDRAVIAKILRSSKYRVTTVESATRALELLCLGLVPNVNMIITDYWMPGMTGYELLKRV
KESSQLKEIPVVIMSSENVPNRISRCLEEGAEDFLLKPVRPSDVSRLCSRIR*
>OsRR7 A-type response regulator, Cytokinin signaling
MRVPAAVTGGCGCGVDGGGGCCRGGGKLADWEEGKDDEMKSVVVKGWTRMAQVVPL
HDNASAEDDDDDEEDDDEDDDDDDDEDDEEEAAPPYVMAVDDSSVDRAVITALLRRSK
YRDSGKRALEILGSEPNVSMIITDYWMPEMTGYDLLKKIKESSELKQIPVVIMSSENVPTRI
SRCLEEGAEDFLLKPVRPADISRITSRMLQ*
>OsRR8 A-type response regulator, Cytokinin signaling
MSSPHVLVVDDTHVDRHVVSMALMRHNVRVTAVESVMQALMFLDSEHDVDMIVSDYC
MPDMTGYNLLMEVKKSPKLAHLPVVIASSDNIPERIRKCLDGGAKDYILKPVKIVDVPRI
MKYI*
>OsRR9 A-type response regulator, Cytokinin signaling
MAVAIEAPFHVLAVDDSLPDRKLIERLLKTSSFQGLAFVCVVTTVDSGSKALEFLGLHDHE
DSPISTQSDQQEVAVNLIITDYCMPGMTGYDLLKKIKESSYLRDIPVVIMSSDNIPSRINRCL
EEGADEFFLKPVRLSDMSKLKPHILKSRCKEHYQQEQNLQSNSESNNSSNPTSENSSSSTSS
NSHKRKAVDEEILPHTIRPRHS*
>OsRR10 A-type response regulator, Cytokinin signaling
MAVAIEAPFHVLAVDDSLPDRKLIERLLKTSSFQVTTVDSGSKALEFLGLHDHEDSPISTQS

DQQEVGVNLIITDYCMPGMTGYDLLKKIKESSYLRDIPVVIMSSDNIPSRINRCLEEGADEF
FLKPVRLSDMSKLKPHILKSRCKEHYQQEQHLQSNSESNNSSNPTSENSSSSTSTNSHKRK
AVDEEILPHTIRPRHS*
>OsRR11 A-type response regulator, Cytokinin signaling
MSSIGAGAGGAVVGAAVAAVAVGGGAPPHVLAVDDSSVDRAVIAGILRSSRFRVTAVDSGK
RALELLGSEPNVSMIITDYWMPEMTGYELLKKVKESSKLKKIPVVIMSSENVPTRISRCLE
EGAEDFLVKPVRPSDVSRLFSRVLP*
>OsRR12 A-type response regulator, Cytokinin signaling
MSSPHVLVVDDTLVDRHVVSMALMRHNVRVTAVESVMQALMFLDSEHDVNMIVSDYC
MPDMTGYDLLMEVKKSPKLAHLPVVIASSDNIPERIRKCLDGGAKDYILKPVKIVDLPRIL
NYI*
>OsRR13 A-type response regulator, Cytokinin signaling
MSSPHVLVVDDTHVDRHVISMALMRHNVRVTAVESVMQALVFLDSEHDVNMIVSDYCM
PEMTGYDLLMEVKKSPRLVHLPVIIASSDNIPERIRKCFDGGAKDYILKPVKIADVPRILNY
I*
>OsRR21 B-type response regulator, Cytokinin signaling
MAPVEDGGGVEFPVGMKVLVVDDDPTCLAVLKRMLLECRYDATTCSQATRALTMLREN
RRGFDVIISDVHMPDMDGFRLLELVGLEMDLPVIMMSADSRTDIVMKGIKHGACDYLIKP
VRMEELKNIWQHVIRKKFNENKEHEHSGSLDDTDRTRPTNNDNEYASSANDGAEGSWKS
QKKKRDKDDDDGELESGDPSSTSKKPRVVWSVELHQQFVNAVNHLGIDKAVPKKILELM
NVPGLTRENVASHLQKFRLYLKRIAQHHAGIANPFCPPASSGKVGSLGGLDFQALAASGQI
PPQALAALQDELLGRPTNSLVLPGRDQSSLRLAAVKGNKPHGEREIAFGQPIYKCQNNAY
GAFPQSSPAVGGMPSFSAWPNNKLGMADSTGTLGGMSNSQNSNIVLHELQQQPDAMLSG
TLHSLDVKPSGIVMPSQSLNTFSASEGLSPNQNTLMIPAQSSGFLAAMPPSMKHEPVLATS
QPSSSLLGGIDLVNQASTSQPLISAHGGGNLSGLVNRNPNVVPSQGISTFHTPNNPYLVSPN
SMGMGSKQPPGVLKTENSDALNHSYGYLGGSNPPMDSGLLSSQSKNTQFGLLGQDDITG
SWSPLPNVDSYGNTVGLSHPGSSSSSFQSSNVALGKLPDQGRGKNHGFVGKGTCIPSRFAV
DEIESPTNNLSHSIGSSGDIMSPDIFGFSGQM*
>OsRR22 B-type response regulator, Cytokinin signaling
MLLGALRMEERKGLMGRERDQFPVGMRVLAVDDDPVCLKVLETLLRRCQYHVTSTNQA
ITALKLLRENRDMFDLVISDVHMPDMDGFKLLELVGLEMDLPVIMLSVNGETKTVMKGIT
HGACDYLLKPVRIEELRNIWQHVVRRKFGNRERNNLDFSKECNKPQSADTDHGPYQPTC
GSSDQNGRSSRKRKELHGEDDDEGDDNDYQENDEPSAAKKPRVVWSVELHRKFVAAVN
QLGIDKAVPKRILELMNVEKLTRENVASHLQKYRLYLKRLGAVASQQASIVAAFGGRDPSF
LHIGAFEGLQSYQPFAPSAALPSFNPHGLLTRTSAAAAFGLQELAAPSSTIQTSTGNVTVGH
CLEENQQANLAQGLTAAIGQPQLQQNWIHQEGNGLSDVFSGSSLTNTLSSTLQRVPSSSLP
PQELLECKQAKVSMPPSIRIPPSSSALLERTLGVSTNLGDSSISQQGALPIDGGFSADRLPLH
SSFDGAVATKLDTSLAASQREIGQQGKFSVSMLVSPSDNLALAKNAKTGASSSGSTIILPLD
TARHSDYLQFGGASNSLQKMDGQKQDHIQSSNIIWSSMPSTQLPSDTQIHNTQNQRLDSG
SFNHNIGAHLADQTNASASILPQMKFDTRISEEKMKQKNTYDLGSSKLQGGFNSSGCNFD
GLLNSIIKVEKDDLPFMDNELGCDLFPLGACI*
>OsRR23 B-type response regulator, Cytokinin signaling
MRAAEERKGVVPAARRRDQFPVGMRVLAVDDDPVCLKVLETLLLRCQYHVTTTNQAAI
ALKMLRENRDMFDLVISDVHMPDMDGFKLLELVGLEMDLPVIMLSVNGETKTVLKGITH

GACDYLLKPVRIEELRNIWQHVIRRKFSTRDRANLDFYEECNKPPNADSDHVHGHVTCGS
PDQSGRPSKKRKEYCSEEEDEGEVNTQDIDDPSAPKKPRVVWSVELHRKFVAAVNQLGID
KAVPKRILELMNVEKLTRENVASHLQKYRLYLKRLSAVASQQVSIVAALGGRDPFLHMGG
FEGLQGYQAFTSSAALSSFTPHGLLNSPRNNPAALGTQGVPASKSIQTMSGSHTLSHSIND
ANKYHLSLPGNQKGNLGQGLATSLGQTQMQQKWIHEETDDLSTILSGNGLSNGMSGTLQ
SVTSSPLLPQELAECTQAKIVSQPSIRTSSVSSEHIEGAVGVSSGLLESRVSQQSTIPLSGFSA
NGLLIHGSFNNTCANKLGGTSSSCAPARSSNDLMVARDTKGGASSFGGAMLLPPDTEQKY
LNFGGGNGLKQKFDDRTADSLFDLKFVWSSVPSSQLASNIGAHHAMSQRWNNSSSNSSNI
GARMIGQATSSGSTVIPQMKTDFLVSGDMAMPKNASDLSIPKLQSELSSSSCSFDGLLNSIV
KVEKDDVTFSDDLGCGDFYSLGACI*
>OsRR24 B-type response regulator, Cytokinin signaling, Rice meiosis and fertility
MTVEERQGRVGGHGVSGGGGGRDQFPVGMRVLAVDDDPTCLKILENLLLRCQYHVTTT
GQAATALKLLRENKDQFDLVISDVHMPDMDGFKLLELVGLEMDLPVIMLSANGETQTVM
KGITHGACDYLLKPVRLEQLRTIWQHVIRRKNCDAKNRGNDDDAGQKAQGMNNEGESI
GANRNKRQSRKSRDENGDDGDDSDENSNENGDSSTQKKPRVVWSVELHRKFVAAVNQL
GIEKAVPKKILDLMNVENITRENVASHLQKYRLYLKRLSTDASRQANLAAAFGGRNPAYIN
MNSFGNYNAYGRYRTVPTAGHTQANNILTRMNSPSAFGVHGLLHSQPIQLGHAQNNLSTS
LNDLGGLNNGNMIRGAQMSTILTGPSGNSFPNISNGAPLATANRSLQPLESSNQQHLSRVH
SSSADPFSTLVGESPQFPDLGRTTNTWQTAVPSNIQDRGHNDNMSQATLHMNGPKIEPVSS
FTSSNQIPLLGNEMQGQVASLASNVPIAFNQDTSPFNYGSSTNSRDMLNNSHVFSNSSINTS
LPNLSLDNPAVPRQTLDRGNTGIVSPMQDGRIHHQAVSNQLNYNDDLMRTTGLQRGLSGG
LDDIVVDMFRPDREDDGVPYIDGDWELV*
>OsRR25 B-type response regulator, Cytokinin signaling
MAATQATAARKFPEGLRVLAVDDSPVCLMLLEALLRRCKYQPTMTRDAATALRMLRERP
GDFDLVISDVHMLDMDGFKLLELIGLEMDLPVIMQSANGELETMMKGVTHGACDYLVKP
VSLKDIQNIWQHVWRKRKLDIRNHNGGYNDGGELVGATRTKRKYTRKMRNDGDNYGE
NKENMDSTLKRQRVVWTPELHRDFVIAVHELGVDRAVPRKILRMMKVDYMTRENIASHL
QKYRLYLKRISTQTGMDPDQFPEKWKYMNELDALKNYCENGRYRLTPAIASSSSSNPFAR
MNSASALATNGFLPTHSVQLKNSQRNMAMGTVGHGGSPGNNPVFQPLQNSSNARKCFPS
GPSGSSFANISNGLVLDTDDSGSSYAGMFCKSMWETSNGSPSCHSGNSSANKSNNGVSAP
ANQFQVQSKFGFSALANQFPVQSNCGFSAPANQYQVQSNGGFSVPANQFPVQSNGEFLAP
TNQFPVQYPEVNNQPLVQMNQSSTNHFSTIGNDYQFPDLANCSKYWQPTAPSMFPDLGH
NDGTSFRPSQANIANINQLSSFAASSGQEPMFGDELHGQMSPIMSTISLSDFDDQMGSFNIG
NDTSPAEMMHDNFSLGSDSNISSSTPTDSSFGSTFPDFHLDSPEMPAQMLNGGDEDGILLP
VLDDTVDQQDLFDQLDENNGNFQSNSVVKALVFAS*
>OsRR26 B-type response regulator, Cytokinin signaling
MDATAFPYGLRVLVVDDDPTWLKILEKMLRKCSYEVTTCGLARVALDILRERKNKFDIVIS
DVNMPDMDGFKLLEHIGLEMDLPVIMMSIDGETSRVMKGVQHGACDYLLKPVRMKELR
NIWQHVYRKKMHEVKEIEGNDSCDDLQILRNSFEGLDEKSLFMRSDSDTMRKRKDVDK
DHADQESSDGNTVKKARVVWSVDLHQKFVNAVNQIGFDKVGPKKILDLMNVPGLTREN
VASHLQKYRLYLSRLQKQNEERILGAARQDFSHKGTSENLNLRSSFQEQPSNIANGYPHAS
QNIQTQANMLDSQLEDTKSTVPLPVPDKKRTLASDAADSQNVTSASSLGGVLSFKSMPVN
QDRKPSETMILECQAWTGGIPSKQFMQYPKHNHERCDLLGDYSCLPKPDLEHPVGPSNLY
APPPLISMSCGMEGDARDFSDVKPAIMDCIKSLSPALTCTVDSVSVQLSDSVVTSIDGDLKS

SGVDGLPSIKDCCLDQTNSQGSLRPSQEPSIIGSTELASLPEDLPSYPLHGVSLENIGLSSIDL
LNYSDAMILSGLQSNWYDDLEFSSEMMDYPSIDECLFASS*
>OsRR27
MAENNGAVPPGCKLPAGGFFGRLHVLVVDDDAAYLEELKLMLLLAGYAVTGKTTAEEAL
KEVDQNPEDYFHIVMTDVHMSGMDGFDLLHRINGRVPVIMFSEGEDVVMVMRTVMNGA
CDYMVKPMTSEAIKFIWKHVLRWRLSALPANASSSLQPSDHLAAALAAVAPPPPPAVQLPA
APAQAGNRDGEAHEEAELSTQPPALVPSGVQEAAAAVWSSRGDGQEAPPPAVAAAAKAPS
KKRGASEVSDRGSNNLEATTGRKKVRTRFTWTTVSHTSFVRAYEQLKDQEGPKKIKQLM
ELDGIFVTKTQVSSHLQKYRSWLENERKKEEATSSSPCNPLSYTNCLDRGYSTWKQSSVIT
EGQQSSSFSGRPIHSMATSNGCLTTTDTQAGNYVGVGAKEIENFISSHQRSLGTAIGQESTI
EQASLHSEITSVSRDAHENGNSQARGSAMSNGTSGTRGVLVTNENLLHVVSASLPSNMG
QPTQPSQSFCTNELAANYSIISDQNPGTSHPTSSSAINNQNSKTQEMSVSQTVELGCGNDV
MLDWPELVGLEDQLDNDVLMNSFFDGDLLQQGVVTAIDGTQEMLAFDSTGDLGSVPPRG
LNNEIASHENTNGKNGASSGP*
>OsRR28
MAQNEGIPNGTLSAMVIDEDKCHADSTCSMICTQLNFCVTVFTSPIKALDFLQNQAEGVH
LVLADVQMEEMNGFEFLKVARELHKSIQVIMMSTETTIYTMKRCVQLGAQILVKKPLDVV
TIQNLWQHLDIKVLKMEKIKDMLQGVGDKSTCANEMNSFPENQKDGTKRKYYLMWTPH
LQKKFLHALEILGEGQISLMIMDVDNIDRKQISTHLQKHRLQLKKKLSKASFTKGSNEDTS
NPSAKNHLTCRTMTLQPHPYTNQPAETTMQIHSEDVEHDDVYDAMRRALQDGTAFDESK
YSSDPFSNEDEDVVGDGYADKANAIDSSGDHYQVAVVLTTPHNVDYTQEIMNKVTTSDD
VQVTRGGKATVSRLVDYSDSDSD*
>OsRR29 LOC_Os04g28130.1
MAQKEGLPAGRLSAMVIDEDKCHADSTSYMLSAELNFSVTVFTSPIKALDFLQNHAEGV
DLVLADVHMEEMNGFDFLKVARELHKSIQVIMMSTETTMYTMKRCVKLGAQFLVNKPL
DAGTIKNLWQYVDLKVLRMEKIKDLLQGIGDESTCANETNSLAENPKNDTKKKYYLMW
TPHLQKKFLHALQILGKDASPKNIKKIMGVDNIDCRQIAAHLQKHRLRLTKDLKKASFTT
DTSKDESNSRIGPAESHHVCRNASTLQPRSNTQPTETTMQILSEDAEYDDVYAAMRRALQ
YGIVFDESKHSSDPSGDEDEQVVVGGDQDGCANEANDIDSSGDHHQVAAVVTKPCNANA
SQEIINKMTNSDGMQATKGSKAAVFRLVDYSESDSD*
>OsRR30 B-type response regulator, Floral inducer to promote short-day (SD) flowering pathway
MDHRELWPYGLRVLVIDDDCSYLSVMEDLLLKCSYKVTTYKNVREAVPFILDNPQIVDLV
ISDAFFPTEDGLLILQEVTSKFGIPTVIMASSGDTNTVMKYVANGAFDFLLKPVRIEELSNI
WQHIFRKQMQDHKNNNMVGNLEKPGHPPSILAMARATPATTRSTATEASLAPLENEVRD
DMVNYNGEITDIRDLGKSRLTWTTQLHRQFIAAVNHLGEDKAVPKKILGIMKVKHLTREQ
VASHLQKYRMQLKKSIPTTSKHGATLSSTALDKTQDHPSRSQYFNQDGCKEIMDYSLPRD
DLSSGSECMLEELNDYSSEGFQDFRWDSDKQEYGPCFWNF*
>OsRR31 LOC_Os08g35650.1
MEDQLSFFPGGLRVMPVDGDTKNTRTATKTLSTLHYSLVATHTTASAGLCTLSSDNMTDV
QTVLCDVKKVVSSGFDFRRVVETEHHIPVIYLLSTTEPEQMVAGEDTEFLNHLLLKATYIV
RKPLDQATMAQLWRVVAWRRCCLEERIPRDSMDDIAAHAGVVGKDGNDNDVIIIEEPQV
HFKVVRSRGSRKRQLTINVDSGSSDGADANPRQKLEHKKDAKGPLGQHVASHLQPQEYC
TKQQKDLDERRLLSLDSLFLKAILSTLNVSLCNPLILTVPAAFTPQDGMTMNKDKAPMIEL
PFGLPVDDFLVGQTAYGSAGPSIGAPDDNDDDAAMYAYTSALNNNAAVGSLMVPPIESTF

TIIDPIVGTKGEGSVPVVVVSEDQNNAVAAIEATAPNNAELFMMPEQVAVDAPVDVEEGIM
FSLESLLGLDEDMIPMEDAGGEATDDSLNIKEGGMEIGWDLDLDYILMNNTNEFAFLDDM
AWIE*
>OsRR32 LOC_Os08g17760.1
MEDMLSFFSSGLHVMLVDDDTKNTRTATKTLSMLHCPVVSTHTTACAGLRTLSGDNMLD
VQTVLCDVSKVVSSGFDFRRVNETEHQIPVIYQERVPGDSMDDIAAHAGAGGEDGNDDD
VVVIEEPQVHFKLVRSRGSRKRQLTINVDSGSSDSAAFKYAVWLIISIRSTDIDLSDANQRK
KIEHMNDAKGPVGQHVASHLQLPAQEYCTKQQKDLDERRLISSDSLFLKAIFPTLNVSPSS
PLILAGGAGPSCIPTTTIAGSRTAAPFQVPVFQQQPSGTTVISFSNTAVQAPIGNAFISFNNAA
SPAATGNTVISFNNIAAPAAMQVPAMRQRLSGGVQPDAPQQRLYMGPFSYQGPPPPPTMR
NHINIVPTAFIPRVGMTVNIGKAPMIELPFGVPVDDFLVGETAYGGAGPSIGAPSNDAAVAY
AYTGALNNNTAVGSLMAPPIDEPTFTLTDPIVGTKGEGVVHIVITSEDQNALAAVEAGAPN
NAEPFMMPDQVDLEEDIMFSLESLLGLDEDMIPMEDAGGEAAEGSLNIGEGGMEIGWDL
DLDDILMNNTNEFAFLDDLAWIE*
>OsRR33 Signal transduction response regulator, receiver region domain containing protein
MDQARISFFPDGLRVMIIDDDAKAVRRATATLSQLQYAVVATHSTASAGLRALSGDNVVEI
QAILCDVHKVVSSGFDFRRVVESELRIPVIYLLSKMEEEDMVAGEDAEFLNHLLLTATYIV
RKPLNPTVMARLWRVVAWRMYCLEERIQANVAANAGAGGEDDDDDDDVVIVEEPQVHF
KVVRRTSGGSRKRQLTINVVDDGNRGSGSGGGGGGGADANPTRILQHITSNLQEFRTKHQ
KKDMAIERPLISSDSMFLKAILPTLKISPCNPLTLTGGIGSSSVAAEAFAGGSSSAAPLQIPVF
QQQSTGNGNTVISFSNNASPMAMRAPTDNTMISFNNVSAAPVANAVISFSNISRSAAMQAP
AARGQHLSGDVQQLDFPQQKLYFGPFSYQGPPPPSMHNHINLLPPTSSPVTCSMDKGKVPI
IELPYGMPVDDFLVSQTTYGGAGPSIGATDAAAAAYPYTDAPSNNVATGCLMVPPMGPAF
SITEPTVVAQGEGTGTGVDAGTSEKNAIVEAPNNPAPLMVLDQVAADAAMDVQEDIMFSL
ESLLGPDYDLLPMEDVSAPDTAAAGDAAGGSLDGEEGGMDIGWDLDLDDILVENVNDFA
FLDNLAGSE*
>OsRR41 Signal transduction response regulator, receiver region domain containing protein
MARKMIRVLLVEDEEINRVVARAALKAAGGGDVVDEAENGEVAVQRVRDAAAPYDLVL
MDKQMPVMDGHEATRRIRGMGVTTPIVAVSSDGLPADVDAFITAGADDFTSKPLSKEKLG
VILAKFRLA*
>OsRR42 Hypothetical conserved gene
MAFQTQGSNLRALLVEDIKVNRMILSQMLRKFQVETTVVQNGKEAVELFLGGETFDIVLT
DNLMPIMTGPEAISKIRAMGATDVMIVGVSVDANSMEEFKDAGADLCVPKLKLEILEHIL
QETRSKKNKSSA*
