# Supplementary material for: Improvement of Rice Agronomic Traits by Editing Type-B Response Regulators
Source: Int J Mol Sci. 2022 Nov 16;23(22):14165. doi: 10.3390/ijms232214165 (PMC9698459; doi:10.3390/ijms232214165)
Supplement: Supplementary file 1 [file ijms-23-14165-s001.zip › ijms-1986700-Supplementary/Supplementary 2.pdf]

Primers used in this study

Primer	Sequence (5′ To 3′)	
21U3F	ggcACGGCGTCTCCTCCCAAGCCG	Vector construct
21U3R	aaacCGGCTTGGGAGGAGACGCCG	Vector construct
21U6aF	gccgCCAGCACCTTCATCCCCACG	Vector construct
21U6aR	aaacCGTGGGGATGAAGGTGCTGG	Vector construct
22U3F	ggcAGAAGTGCTGAACTAGAAGG	Vector construct
22U3R	aaacCCTTCTAGTTCAGCACTTC	Vector construct
22U6aF	gccGCTCCAATATGCAAGAAGGA	Vector construct
22U6aR	aaacTCCTTCTTGCATATTGGAG	Vector construct
23U3F	ggcaCTATCGCGCTGAAAATGCTG	Vector construct
23U3R	aaacCAGCATTTTCAGCGCGATA	Vector construct
23U6aF	gccGTATTGAAGGGGATAACCCA	Vector construct
23U6aR	aaacTGGGTTATCCCCTTCAATA	Vector construct
24U3F	ggcATCAACACAGAAGAAGCCAA	Vector construct
24U3R	aaacTTGGCTTCTTCTGTGTTGA	Vector construct
24U6aF	gccGAATGTAGAGAACATCACCA	Vector construct
24U6aR	aaacTGGTGATGTTCTCTACATT	Vector construct
25bU3F	ggcaGCTTGAGGAGGCAATGGCTG	Vector construct
25bU3R	aaacCAGCCATTGCCTCCTCAAGC	Vector construct
25bU6aF	gccGCATTTGATGAGTTCTGCAG	Vector construct
25bU6aR	aaacCTGCAGAACTCATCAAATG	Vector construct
26U3F	ggcACATCACTGTGGAAACAGAA	Vector construct
26U3R	aaacTTCTGTTTCCACAGTGATG	Vector construct
26U6aF	gccGCAGATCAGGAGTCAAGCGA	Vector construct
26U6aR	aaacTCGCTTGACTCCTGATCTG	Vector construct
27U3F	ggcaGCAGCGGTATGGAGCAGCCG	Vector construct
27U3R	aaacCGGCTGCTCCATACCGCTGC	Vector construct
27U6aF	gccGCAGCCAAGCGATCACCTGG	Vector construct
27U6aR	aaacCCAGGTGATCGCTTGGCTG	Vector construct
28U3F	ggcaGTAGTCTGCATGCCAACCAA	Vector construct
28U3R	aaacTTGGTTGGCATGCAGACTAC	Vector construct
28U6aF	gccgTTGAGCATGTAGAATCAGCA	Vector construct
28U6aR	aaacTGCTGATTCTACATGCTCAA	Vector construct
29U3F	ggcaGGTGCTGGCAGATGTCCACA	Vector construct
29U3R	aaacTGTGGACATCTGCCAGCACC	Vector construct
29U6aF	gccgTGTAGCTTGTAGAATCAGCA	Vector construct
29U6aR	aaacTGCTGATTCTACAAGCTACA	Vector construct
30U3F	ggcAGATCACAAGAACAATAACA	Vector construct
30U3R	aaacTGTTATTGTTCTTGTGATC	Vector construct
30U6aF	gccgAGGTCAGCCTGGACTTTCCG	Vector construct
30U6aR	aaacCGGAAAGTCCAGGCTGACCT	Vector construct
31U3F	ggcATGTCGTCCATGGAGTCACG	Vector construct

31U3R	aaacCGTGACTCCATGGACGACA	Vector construct
31U6aF	gccgCCTCTGCGATGTCAAAAAGG	Vector construct
31U6aR	aaacCCTTTTTGACATCGCAGAGG	Vector construct
32U3F	ggcaGTTCGGTCTCGTTGACACGG	Vector construct
32U3R	aaacCCGTGTCAACGAGACCGAAC	Vector construct
32U6aF	gccGCTATCGTGGCCCAGTCGAG	Vector construct
32U6aR	aaacCTCGACTGGGCCACGATAG	Vector construct
33U3F	ggcaCCACCTTGTGGACATCGCAG	Vector construct
33U3R	aaacCTGCGATGTCCACAAGGTGG	Vector construct
33U6aF	gccGTTTATAGTCGATCATTCGA	Vector construct
33U6aR	aaacTCGAATGATCGACTATAAA	Vector construct
U-F	CTCCGTTTTACCTGTGGAATCG	Vector construct
gRNA-R	CGGAGGAAAATTCCATCCAC	Vector construct
Uctcg-B1’	TTCAGAggtctcTctcgACTAGTGGAATCGGCAGCAAAGG	Vector construct
gRctga-B2	AGCGTGggtctcGtcagGGTCCATCCACTCCAAGCTC	Vector construct
Uctga-B2’	TTCAGAggtctcTctgaCACTGGAATCGGCAGCAAAGG	Vector construct
gRcggt-BL	AGCGTGggtctcGaccgACGCGTCCATCCACTCCAAGCTC	Vector construct
Hpt757F	GGGCGTCGGTTTCCACTATC	Transgenic analysis
Hpt757R	GGGAGTTTAGCGAGAGCCTG	Transgenic analysis
Cas9F	CACGAGGTCCGACAAGAACA	Transgenic analysis
Cas9R	ACCTTGCGAACAGTAGCGAA	Transgenic analysis
21TF	GGCAGCAGTAGCAGGCATCA	Target site sequencing
21TR	AGCAAATCAACCCACGGAGC	Target site sequencing
22TF	GGATAAATTGAACGAGGTTCC	Target site sequencing
22TR	TCACTTGGCAGTTGAGTGCTT	Target site sequencing
23TF	TCAAGGTGCTCGAGACCCTG	Target site sequencing
23TR	TCTGAATCCGCATTTGGTGG	Target site sequencing
24TF	AAGGCACAGGGAATGAACAATG	Target site sequencing
24TR	ACCGTTTAAGGTACAGCCGATA	Target site sequencing
25TF	TGCGGATGATGAAAGTGGAC	Target site sequencing
25TR	GCAGACGAGTTGCCAGAATG	Target site sequencing
26TF	ACATGCCTGATATGGATGGTT	Target site sequencing
26TR	TTGCTCTTTCGGAATGAGATT	Target site sequencing
27TF	GGTGATGAGGACGGTGATGAA	Target site sequencing
27TR	TGCTCGTATGCCCTGACAAAC	Target site sequencing
28TF	GAAATTATCATACGCAATGGTC	Target site sequencing
28TR	CAACTTTAAGGAACTCAAAGCC	Target site sequencing
29TF	CTCCCTGCTGGTAGGCTTAG	Target site sequencing
29TR	TGACGCACCTCTTCATTGTG	Target site sequencing
30TF	TCACAATGGGCTGCTCTAGT	Target site sequencing
30TR	TTCTTTGGAACTGCCTCTGC	Target site sequencing
31TF	TTTTCTAGTGGTGGCAACGC	Target site sequencing
31TR	TCAGAAGAACCTGCACCGTC	Target site sequencing
32TF	TAGACGTCCAGACTGTCCTCTGCGA	Target site sequencing

32TR	GGTAACGGCTAATTTCATACGAG	Target site sequencing
33TF	CACATCAACCTACTCCCGC	Target site sequencing
33TR	AGCACACATCCCCTCTCTC	Target site sequencing
qHd3a-F	CTTCAACACCAAGGACTTCGC	qRT-PCR
qHd3a-R	TAGTGAGCATGCAGCAGATCG	qRT-PCR
qRFT1-F	TGACCTAGATTCAAAGTCTAATCCTT	qRT-PCR
qRFT1-R	TGCCGGCCATGTCAAATTAATAAC	qRT-PCR
qMADS14F	TGGGACCAGACACAACCTCA	qRT-PCR
qMADS14R	CCTGCTGCTACATCCTCTAT	qRT-PCR
qMADS15F	CGTCGTCGGCCAAACAG	qRT-PCR
qMADS15R	TGACTTCAATTCATTCAAGGTTGCT	qRT-PCR
qUbiquitin-F	AACCAGCTGAGGCCCAAGA	qRT-PCR
qUbiquitin-R	ACGATTGATTTAACCAGTCCATGA	qRT-PCR
